# Conservation of Indigenous Vegetables from a Hotspot in Tropical Asia: What Did We Learn from Vavilov?

**DOI:** 10.3389/fpls.2016.01982

**Published:** 2017-01-04

**Authors:** Svein Ø. Solberg, Yu-Yu Chou

**Affiliations:** Theme Germplasm, World Vegetable CenterTainan, Taiwan

**Keywords:** biodiversity, crop wild relative, geography, genetic resources, genebank, horticulture, vegetable

## Crop diversity and geography

Conservation biologists have allocated an Indo-Burma biodiversity hotspot among 34 regions around the world especially rich in plants, animals, and other species (Myers et al., 2000). More than 13,500 different vascular plant species, of which 7000 are endemic, have been detected in this hotspot (Tordoff et al., 2012). Ninety years ago, the Russian scientist Vavilov (1926) pointed to the richness of cultivated plant species and their crop wild relatives in certain areas around the world, of which the *Tropical Asia Center* was one of eight. Later, Zeven and Zhukovsky (1975) applied the term *Indochinese—Indonesian region of diversity* for the area, which by then had been divided by various authors into an Indochinese region including Myanmar, Laos, Cambodia, Thailand, Bangladesh, and parts of Northeastern India and Southern China, and a more southern region including Malaysia and Indonesia (Darlington, 1956; Li, 1969; Schery, 1972; Janick, 2002; Nandwani, 2014). Rice (*Oryza* sp.), mungbean (*Vigna radiata*), gourd species, and indigenous vegetables were among crops that Zeven and Zhukovsky (1975) listed in the Indochinese region of diversity.

Vavilov noted the importance of collecting different varieties, and the wild relatives, of cultivated plants as genetic resources for use in crop improvement. In 1935, he claimed that for plant breeders there was no other choice but to become geographers (Vavilov, 1935). At that time, the genepool available to plant breeders was relatively narrow. To adapt crops to a wider geographical zone and to move forward in breeding, Vavilov started collecting germplasm worldwide and conducted expeditions to India, China, and other locations. He also received samples of leguminous species from Myanmar. In a later overseas collection mission in Myanmar, Kobylyanskaya, and Chepurin from the Vavilov Institute collected 201 accessions of cereals and legumes from the central and south parts of the country (Loskutov, 1999). More recently, Japanese scientists have conducted missions to Myanmar to collect wild rice (Uga et al., 2004).

As global populations increase and climates change, a greater diversity of genetic resources is needed for crop improvement. Agriculture needs to produce more food under more unpredictable climatic conditions (McCouch et al., 2013; Nierenberg, 2013; Betts and Hawkins, 2014). Genetic resources have indeed been collected; according to the UN Food and Agriculture Organization (FAO, 2010), rice is well-represented, with more than 770,000 accessions in genebanks around the world. Horticultural crops such as vegetables tend to be highly underrepresented in the conservation system, especially considering their nutritional importance (Yang and Hanson, 2009). Here we demonstrate, despite 90 years of awareness, how indigenous vegetables are poorly represented in the global conservation system and argue for the importance of safeguarding agrobiodiversity in its centers of diversity. We use tropical Asia as a case area but narrow our focus to Myanmar and indigenous vegetables relevant for this country.

## Myanmar and target crops

Myanmar is within a recognized region of diversity of several cultivated plants. The country has a tropical monsoon climate, a distinct wet and warm summer and a dry and relatively warm winter. The current population is 51.7 million. Myanmar is a member of the Association of Southeast Asian Nations (ASEAN) and recently joined the ASEAN economic community. Agriculture is important and generates more than 20% of export earnings and employs more than 60% of the labor force (MOAI, 2015). Rice is cultivated on two thirds of the arable land; around 1 million tons of rice are exported annually. Pulses are the second most important crop, grown on 4.5 million ha, and include species such as black gram and mungbean (green gram). Vegetables are grown on around 0.8 million ha, principally in the highlands but also in household gardens or small-scale market gardens throughout the country. The conservation system of plant genetic resources in Myanmar is organized by the Department of Agricultural Research with national seed and field collections and a common information system maintained at the Myanmar Seed Bank and funded by the government of Japan (FAO, 2016).

We were interested in vegetable crops that Zeven and Zhukovsky (1975) linked to Tropical Asia and Myanmar as region of diversity. They used the term “S. Asia” for this area in contrast to SE. Asia, which was used for the more southern area including Malaysia and Indonesia. Typical medicinal plants, bamboo species, and starch plants were not included, although some would call them vegetables. In addition, a second list of vegetable crops was made based on those regarded as important indigenous vegetables in Myanmar today by national agricultural experts from the Seed Bank Myanmar, Department of Agricultural Research, or as presented in a publication of important food plants in Myanmar (Watanbe et al., 1999). The two lists were merged and more than 30 target species were identified (Table 1).

**Table 1:** **Targeted species, their region of diversity, number of occurrences, and number of conserved accessions globally and with Myanmar as country of origin**.

**Scientific and common name**	**Region of diversity^A^**	**No. of occurrences GBIF (2016)**		**No. of accessions GENESYS (2016)**	
		**Global**	**Myanmar**	**Global**	**Myanmar**
**VEGETABLE SPECIES WITH DIVERSITY CENTER INCLUDING MYANMAR**					
*Vigna radiata* (mungbean)	India, Myanmar	11,879	4	11,953	10
*Wolffia arrhiza* (spotless watermeal)	India, Myanmar	10,223	0	0	0
*Momordica charantia* (bitter gourd)	T Asia	6044	5	451	0
*Ocimum basilicum* (sweet basil)	T Asia	3083	0	444	0
*Oenanthe javanica* (Chinese celery)	Indochina	3066	2	3	0
*Mucuna pruriens* (Cochichina)	T Asia	2220	6	48	0
*Vigna mungo* (black gram)	India, Myanmar	1929	2	1518	0
*Luffa aegyptiaca* (sponge gourd)	India, T Asia	1583	4	532	1
*Amaranthus tricolor* (amaranth)	T Asia	1248	0	473	0
*Basella alba* (Indian spinach)	T Asia	1051	5	170	0
*Trichosanthes cucumerina* (snake gourd)	T Asia	963	7	46	0
*Neptunia oleracea* (garden puff)	T Asia	937	2	9	0
*Psophocarpus tetragonolobus* (winged bean)	T Asia	839	1	485	0
*Benincasa hispida* (waxgourd)	T Asia	513	1	383	0
*Canavalia gladiata* (sword jackbean)	Myanmar	349	3	28	0
*Sesbania grandiflora* (veg. hummingbird)	T Asia	6	0	114	0
**INDIGENOUS VEGETABLES IMPORTANT FOR MYANMAR**^*B*^ **BUT FROM OTHER DIVERSITY CENTERS**					
*Vigna unguiculata* (cowpea)	Africa, India	37,203	2	19,429	4
*Brassica nigra* (black mustard)	Europe	19,824	0	231	0
*Cajanus cajan* (pigeon pea)	Africa, India	17,439	14	13,590	74
*Sinapis alba* (white mustard)	Europe	14,471	0	538	0
*Phaseolus lunatus* (Lima bean)	C America	9268	1	6088	2
*Solanum melongena* (eggplant)	India, China	5831	7	5487	6
*Hibiscus cannabinus* (roselle)	Africa	5370	3	553	0
*Lagenaria siceraria* (bottle gourd)	Africa	2551	1	1172	1
*Colocasia esculenta* (wild taro)	SE Asia	2266	1	0	0
*Ipomoea aquatica* (kangkong)	No info	1977	7	81	0
*Pachyrhizus erosus* (ice potato)	S America	1310	0	83	0
*Nelumbo nucifera* (lotus)	C Asia	1060	3	2	0
*Sechium edule* (chayote)	C America	900	0	6	0
*Artocarpus heterophyllus* (jackfruit)	India	868	0	15	0
*Luffa acutangula* (sponge gourd)	India	759	3	551	0
*Senegalia pennata* (climbing wattle)		685	18	0	0
*Amorphophallus paeoniifolius* (elephant yam)	SE Asia	570	1	3	0
*Dolichos lablab* (lablab bean)	Africa	565	0	16	0
*Centella erecta* (Asian pennywort)	No info	297	0	0	0
*Combretum indicum* (Rangoon creeper)	SE Asia	260	0	1	0
*Lablab vulgaris* (summer bean)	No info	53	0	0	0

*A grand total number of occurrences in GBIF (2016) was 645,184,811 with 108,787 from Myanmar. A grand total number of conserved accessions in GENESYS (2016) was 2,573,870 with 4092 accessions with Myanmar as country of origin*.

A *Center of diversity according to Zeven and Zhukovsky (1975) (T Asia, Triopical Asia)*,

B *Important vegetables in Myanmar according to Watanbe et al. (1999) and national agricultural expertise*.

The Integrated Taxonomic Information System was applied (ITIS, 2016) to verify the species taxonomic and common names. Each species was then examined by surveying two global databases: the Global Biodiversity Information Facility (GBIF, 2016) and the Global Gateway to Genetic Resources (GENESYS, 2016). GBIF was applied to survey the number of occurrences, applying the scientific names and country filters in the search function. GBIF (2016) occurrences are the recorded number of observations of a species as natural populations, herbarium specimens, seed collects, or others. GENESYS (2016) records reflect the conservation status of plant genetic resources. GENESYS was applied to survey the number of conserved accessions, applying the scientific names and country of origin as filters in the search function. In GENESYS, combined searches were used; for example for mungbean, different taxa, and authors were combined including *Vigna radiata* var. *radiate, Vigna radiata* (L.) R. Wilczek var. *radiate, Vigna radiate, Vigna radiata* (L.) R. Wilczek var. *radiate, Vigna radiata* (L.) R. wilczek, and *Vigna radiata* (L.) R.Wilczek. Filters used are given as Supplementary Material (Table S1).

## Species occurrences

In GBIF (2016), a total of 108,787 occurrences from Myanmar were reported, which was <0.02% out of the global number of 645,184,811. Of targeted species, there were the most records from Myanmar on *Senegalia pennata* (climbing wattle), a wild tree not mentioned by Zeven and Zhukovsky (1975), its leaves are collected and used as a vegetable, followed by *Cajanus cajan* (pigeon pea), a grain legume not included in the Indochina region of diversity (Table 1). Of the species with Indochina as the region of diversity, *Trichosanthes cucumerina* (snake gourd), *Momordica charantia* (bitter gourd), and *Vigna radiata* (mungbean) each had four to seven records from Myanmar in GBIF, which were herbarium specimens or seed collects. These are very low numbers, but they do not necessarily reflect the importance or the presence of the species. Rather, this can be a result of few botanic studies or no reporting from the country to GBIF.

## Conservation status

In GENESYS (2016), a total number of 4092 accessions with Myanmar as country of origin were reported, which was 0.16% out of the global number of 2,573,870 accessions. Targeted vegetable species had from 0 to 20,000 conserved accessions globally, but only with 0–70 accessions from Myanmar depending on the species (Table 1). Mungbean was relatively well-represented in genebanks around the world, but had only a very few accessions from Myanmar; a country mentioned as the primary region of diversity by Zeven and Zhukovsky (1975). In contrast, India is represented by 5154 mungbean accessions in GENESYS. The overall underrepresentation of indigenous vegetables from Tropical Asia is clear. In total, 451 accessions of bitter gourd were reported in GENESYS, none with Myanmar as country of origin, and 81 accessions of the leafy vegetable kangkong (*Ipomoea aquatica*) were reported, also with none from Myanmar. As a comparison, rice (*Oryza* sp.) has 208,447 accessions in GENESYS (and 3543 accessions with Myanmar as country of origin). Among the reported bitter gourd accessions 50 were from Thailand, 40 from Bangladesh, 32 from Cambodia, and 0 from Myanmar—all countries regarded as part of the primary region of diversity. Among the kangkong accessions 27 were from Thailand, 4 from Cambodia and again, 0 from Myanmar. These numbers are all very low. Both bitter gourd and kangkong are common in smallholder gardens in Myanmar and other tropical Asian countries. The crops may likely be present as semi-wild, naturalized populations, as locally cultivated material (landraces), or as modern varieties. GENESYS does not capture all conserved germplasm, as there are collection holders that do not report to the global database. However, surveying the database gives a guideline of the conservation status. A crop such as bitter gourd is used all over tropical Asia, the plant is related to cucumber, and it has several positive health effects, including being good for lowering blood sugar levels (Leung et al., 2009). Kangkong is a nutritious leafy vegetable that can stand wet conditions and flooding (Irfanullah et al., 2011), and thus has potential for climate change adaptation.

## Outlook

Myanmar is experiencing significant economic and political changes (Asian Development Bank, 2012). Its agricultural sector is evolving; seed companies are establishing; and new cultivars are being introduced (Asian Development Bank Institute, 2013; MOAI, 2016). Ongoing changes imply that important plant genetic resources in a recognized region of diversity may be threatened. We have demonstrated that today such genetic resources are sparsely represented in global collections. As gaps have been identified, new collection missions should be conducted. This should be done to safeguard the genetic resources and also to facilitate access for breeding and research as outlined in the *International Treaty on Plant Genetic Resources for Food and Agriculture* (ITPGRFA, 2001). One problem is that most vegetables, and almost all indigenous vegetables, are not explicitly mentioned in Annex 1 of the Treaty. Furthermore, the *Convention of Biological Diversity* and the *Nagoya Protocol on Access and Benefit Sharing* (ABS, 2014) has made overseas collection missions complicated (Cressey, 2014; Maggioni et al., 2015). Ninety yeas after Vavilov published his work on *Centers of Origin*, there are still huge gaps in global collections. Although some major crops have been extensively collected, indigenous vegetables from tropical Asia, and especially from Myanmar, are poorly represented. Such vegetables have great potential to add vital nutrients to human diets (Yang and Hanson, 2009), diversify cropping systems (Subbarao et al., 2005), and increase smallholders' incomes and livelihoods (Ebert, 2014). Indeed, there are challenges ahead: What Vavilov once did is difficult to do today. Now, each country tends to guard its domain; some countries look more inwards than outwards, complicating the access of enterprises and research institutions to genetic material. To paraphrase Vavilov: Plant breeders are restricted from being geographers. Such constraints hinder the best efforts of agriculture to face the grand challenges of our time. International organizations, national policy makers, research institutions, and enterprises should work together and find joint solutions to safeguard and utilize the great potential of plant genetic resources.

## Author contributions

SS had the idea and contributed by co-ordination the writing. YC contributed by database searches and comments.

### Conflict of interest statement

The authors declare that the research was conducted in the absence of any commercial or financial relationships that could be construed as a potential conflict of interest.

## Supplementary material

Table S1 **Scientific names applied as filters in the search function in GENESYS**.
