# Supplementary material for: Conservation of Indigenous Vegetables from a Hotspot in Tropical Asia: What Did We Learn from Vavilov?
Source: Front Plant Sci. 2017 Jan 4;7:1982. doi: 10.3389/fpls.2016.01982 (PMC5209338; doi:10.3389/fpls.2016.01982)
Supplement: Table S1 — Scientific names applied as filters in the search function in GENESYS. [file Table1.docx]

Table S1.

| **Scientific name** | **Filters applied in GENESYS (2016)** |
| --- | --- |
| *Vigna radiata* (L.) R. Wilczek | "Vigna radiata var. radiata" or "Vigna radiata (L.) R.Wilczek var. radiata" or "Vigna radiata" or "Vigna radiata (L.) R. Wilczek var. radiata" or "Vigna radiata (l.) r. wilczek" or "Vigna radiata (L.) R.Wilczek" |
| *Wolffia arrhiza* (L.) Horkel ex Wimm. | "Wolffia arrhiza" |
| *Momordica charantia* L. | "Momordica charantia" or "Momordica charantia l." |
| *Ocimum basilicum*  L. | "Ocimum basilicum" or "Ocimum basilicum l." |
| *Oenanthe javanica* (Blume) DC. | "Oenanthe javanica" or "Oenanthe javanica (Blume) DC." |
| *Mucuna pruriens* (L.) DC. | "Mucuna pruriens" or "Mucuna pruriens (L.) DC.' |
| *Vigna mungo* (L.) Hepper | "Vigna mungo var. silvestris" or "Vigna mungo (L.) Hepper var. mungo" or "Vigna mungo" or "Vigna mungo (l.) hepper" |
| *Amaranthus tricolor*  L. | "Amaranthus tricolor l." or "Amaranthus tricolor" |
| *Basella alba*  L. | "Basella alba" or "Basella alba L." |
| *Trichosanthes cucumerina*  L. | "Trichosanthes cucumerina" or "Trichosanthes cucumerina L." |
| *Neptunia oleracea* Lour. | "Neptunia oleracea" or "Neptunia oleracea Lour." |
| *Psophocarpus tetragonolobus* (L.) DC. | "Psophocarpus tetragonolobus" or "Psophocarpus tetragonolobus (L.) DC." or "Psophocarpus tetragonolobus DC." |
| *Benincasa hispida* (Thunb.) Cogn. | "Benincasa hispida (thunb) cogn." or "Benincasa hispida (Thunb.) Cogn" or "Benincasa hispida (THUNB.) COGN." or "Benincasa hispida" |
| *Canavalia gladiata* (Jacq.) DC. | "Canavalia gladiata (Jacq.) DC." or "Canavalia gladiata" |
| *Combretum indicum* (L.) DeFilipps | "Combretum indicum (L.) DeFilipps" or "Combretum indicum" |
| *Sesbania grandiflora* (L.) Pers. | "Sesbania grandiflora" or "Sesbania grandiflora (L.) Pers." |
|  |  |
| *Vigna unguiculata* (L.)Walp. | "Vigna unguiculata" or "Vigna unguiculata (L.) Walp. subsp. sesquipedalis (L.) Verdc." or "Vigna unguiculata (L.) Walp." |
| *Brassica nigra* (L.) W.D.J. Koch | "Brassica nigra (L.) W. D. J. Koch" or "Brassica nigra" or "Brassica nigra (L.) W.D.J.Koch" |
| *Cajanus cajan* (L.) Millsp. | "Cajanus cajan (L.) Millsp." or "Cajanus cajan" |
| *Sinapis alba* L. | "Sinapis alba" or "Sinapis alba L." |
| *Phaseolus lunatus* L. | "Phaseolus lunatus" or "Phaseolus lunatus l." |
| *Solanum melongena* L. | "Solanum melongena" or "Solanum melongena l." or "Solanum melongena L" |
| *Hibiscus cannabinus* L. | "Hibiscus cannabinus L." or "Hibiscus cannabinus" |
| *Lagenaria siceraria* (Molina) Standl. | "Lagenaria siceraria (Mol.) Standl." or "Lagenaria siceraria (MOLINA) STANDL." or "Lagenaria siceraria (Molina) Standley" or "Lagenaria siceraria" |
| *Colocasia esculenta* (L.) Schott | "Colocasia esculenta" |
| *Ipomoea aquatica* Forsk. | "Ipomoea aquatica forsk." or "Ipomoea aquatica" or "Ipomoea aquatica Forssk." |
| *Luffa aegyptiaca* Mill. | "Luffa aegyptiaca Mill." or "Luffa aegyptiaca" or "Luffa aegyptiaca Mil." |
| *Pachyrhizus erosus* (L.) Urb. | "Pachyrhizus erosus" or "Pachyrhizus erosus (L.) Urb." |
| *Nelumbo nucifera* Gaertn. | "Nelumbo nucifera Gaertn" or "Nelumbo nucifera" |
| *Sechium edule* (Jacq.) Sw. | "Sechium edule (Jacq.) Sw." or "Sechium edule" |
| *Artocarpus heterophyllus* Lam. | "Artocarpus heterophyllus" or "Artocarpus heterophyllus Lam." |
| *Psophocarpus tetragonolobus* (L.) DC. | "Psophocarpus tetragonolobus" or "Psophocarpus tetragonolobus (L.) DC." or "Psophocarpus tetragonolobus DC." |
| *Luffa acutangula* (L.) Roxb. | "Luffa acutangula" or "Luffa acutangula (l.) roxb." |
| *Senegalia pennata* (L.) Maslin | "Senegalia pennata" |
| *Amorphophallus paeoniifolius* (Dennst.) Nicolson | "Amorphophallus paeoniifolius (Dennst.) Nicolson" or "Amorphophallus paeoniifolius" |
| *Dolichos lablab* L. | "Dolichos lablab" |
| *Centella erecta* (L. fil.) Fern. | "Centella erecta" |
| *Lablab vulgaris* Savi | "Lablab vulgaris" |
